# Map-invariant spectral analysis for the identification of DNA periodicities

**DOI:** 10.1186/1687-4153-2012-16

**Published:** 2012-10-15

**Authors:** Ahmad Rushdi, Jamal Tuqan, Thomas Strohmer

**Affiliations:** 1 Department of Electrical and Computer Engineering at the University of California, Davis, CA 95616, USA, and is now with Cisco Systems, Inc., San Jose CA 95134, USA; 2 Department of Electrical and Computer Engineering at the University of California, Davis, CA 95616, USA; 3 Department of Mathematics, University of California, Davis, CA 95616, USA

## Abstract

Many signal processing based methods for finding hidden periodicities in DNA sequences have primarily focused on assigning numerical values to the symbolic DNA sequence and then applying spectral analysis tools such as the short-time discrete Fourier transform (ST-DFT) to locate these repeats. The key results pertaining to this approach are however obtained using a very specific symbolic to numerical map, namely the so-called Voss representation. An important research problem is to therefore quantify the sensitivity of these results to the choice of the symbolic to numerical map. In this article, a novel algebraic approach to the periodicity detection problem is presented and provides a natural framework for studying the role of the symbolic to numerical map in finding these repeats. More specifically, we derive a new matrix-based expression of the DNA spectrum that comprises most of the widely used mappings in the literature as special cases, shows that the DNA spectrum is in fact invariable under all these mappings, and generates a necessary and sufficient condition for the invariance of the DNA spectrum to the symbolic to numerical map. Furthermore, the new algebraic framework decomposes the periodicity detection problem into several fundamental building blocks that are totally independent of each other. Sophisticated digital filters and/or alternate fast data transforms such as the discrete cosine and sine transforms can therefore be always incorporated in the periodicity detection scheme regardless of the choice of the symbolic to numerical map. Although the newly proposed framework is matrix based, identification of these periodicities can be achieved at a low computational cost.

## 1 Introduction

**Figure 1:**
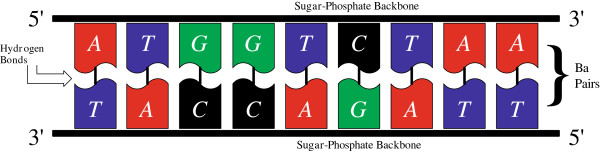
DNA: a straightened helix structure.

Many researchers have noted that the occurrence of repetitive structures in a DNA sequence is symptomatic of a biological phenomena. Specific applications of this observation include identification of diseases [1], DNA forensics [2], and detection of pathogen exposure [3]. Some of these structures are simple repetition of short DNA segments such as exons [4], tandem repeats [5], dispersed repeats [6], and unstable triplet repeats in the noncoding regions [7] while other forms more elaborate patterns such as palindromes [8] and the period-3 component [9-13], a strong periodic characteristic found primarily in genes and pseudogenes [14]. Methods that detect these DNA periodicities are either probabilistic or deterministic. Most of the deterministic techniques rely on spectral analysis of the DNA sequence using the short-time discrete Fourier transform (ST-DFT) [15-17]. The main idea is as follows: given a DNA sequence of length N, numerical values are first assigned to every element in  = {*A*,*C*,*G*,*T*}, where these letters denote the four nucleotides in the DNA, namely the two purines: adenine (*A*) and guanine (*G*) and the two pyrimidines: thymine (*T*) and cytosine (*C*). A typical DNA double helix is shown in Figure 1.

**Figure 2:**
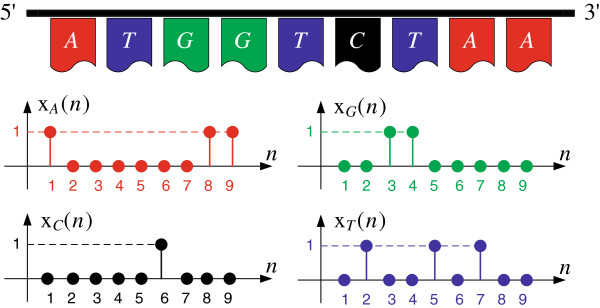
The Voss representation of a DNA segment.

The symbolic to numerical map is clearly not unique, typically has a biological interpretation, and needs to preserve the specific structure of the DNA sequence under study. One such popular map is the Voss representation ↦  = {0,1}, where four binary indicator sequences *x*_*l*_(*n*), *l* ∈ , are generated with 1 indicating the presence of a nucleotide and 0 its absence [18]. An example of the mapping of a single DNA strand to $x_{l}(n),\forall l\in F$ is shown in Figure 2.

Once the DNA symbolic sequence is mapped into numerical version(s), a set of discrete time sequences are generated and are the numerical equivalence of the DNA sequence. These numerical sequences can then by processed using standard signal processing techniques. In particular, the ST-DFT for each elementary sequences can be computed as

(1) $$X_{l}(\mathrm{Rn},k)≜\overset{0}{\underset{m=-M+1}{\sum }}x_{l}(\mathrm{Rn}+m)h(m)e^{-j\frac{2\Pi \text{mk}}{M}},$$

∀l∈F, where *n* is the window starting point, *R* is the amount of window shift, and *h*(*m*) = 1 for −*M* + 1 ≤ *m* ≤ 0 and zero otherwise. If *R* = 1, then, the window slides one nucleotide at a time whereas if *R* = 3, the displacement of the window is on a 3-nucleotide basis. Note that the all-ones function *h*(*m*) does not affect the value of *X*_*l*_(*Rn*,*k*). However, it serves as a place holder for other filters that can be used to replace it, as will be shown in the following section. One popular application of the ST-DFT based technique that has received considerable attention in the past is the identification of the period-3 component using the DNA spectrum, defined for *R* = 3 as follows

(2) $$\begin{array}{ll} \;S(n) & =\underset{l\in F}{\sum }|X_{l}\left(3n,\frac{M}{3}\right){|}^{2}\\ & =\underset{l\in F}{\sum }{\left|\overset{0}{\underset{m=-M+1}{\sum }}x_{l}(3n+m)e^{-j\frac{2\Pi m}{3}}\right|}^{2}. \end{array}$$

**Figure 3:**
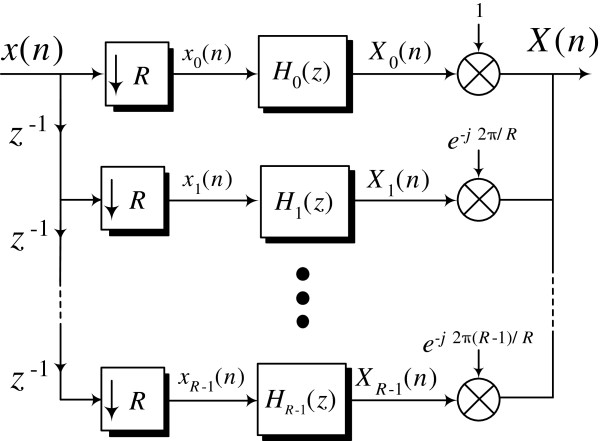
**The Multirate DSP model for general *R*.** The period-3 case is easily obtained by setting *R* = 3.

A number of researchers have advocated the use of the period-3 component to discriminate between coding and non coding regions (see for example [11,13,16,19-23] to name a few) but the subject remains highly controversial as it is successful for certain genes but does not work for others. To better comprehend the underlying reasons behind this disparity in performance, a new multirate DSP model that provides a full understanding of the inner workings of the DNA periodicity has been first proposed in [24], and studied in details in [25]. This model is shown in Figure 3.

This model provides closed form expressions for the DNA spectrum that generalize and unify some of the already existing results in the literature were obtained. One of these expressions in particular clearly shows that the identification of the period-3 component in the DNA spectrum, a signal processing problem, is equivalent to the detection of the nucleotide distribution disparity in the codon structure of a DNA sequence, a genomic problem. The disparity in the nucleotide distribution within the codon structure of a DNA sequence is termed the codon bias. Using this model, the DNA spectrum is completely characterized by a set of digital sequences, termed the *filtered polyphase sequences*. By processing these sequences, signal processing techniques can potentially have an impact on understanding and detecting biological structures of this nature. From a computational cost perspective, the computation of the DNA spectrum using this model does not require any complex valued operations [26]. This finding is rather surprising given the existence of complex multipliers in the proposed DSP model as clearly illustrated in Figure 3. It is shown that the direct computation of the DNA spectrum using (2) requires essentially double the amount of arithmetic operations compared to the DSP model approach.

It is important, however, to keep in mind that the above conclusions and results were obtained using the Voss symbolic to numerical transformation. A fundamental research issue is to therefore determine the sensitivity of the signal processing based method to the choice of the *symbolic to numerical map*. In particular, the core question here is: how dependent are the above results on the Voss representation? Are these results invariant with respect to the other popular maps in the literature? Can we derive necessary and/or sufficient conditions for the invariance of the DNA spectrum to the symbolic to numerical transformation? Is there a general mathematical framework that can help us generate new symbolic to numerical maps for which the DNA spectrum remains essentially the same? These are the type of questions we address in this article and provide answers to. One approach to answer this question was presented in [27], where a novel framework for the analysis of the equivalence of the mappings used for numerical representation of symbolic data based on signal correlation was presented, along with strong and weak equivalence properties. In [28], we attempted to answer the same question starting at the aforementioned DSP model for a limited set of mappings. Our main goal in this study is to de-embed the symbolic to numerical mapping process from the DNA spectrum computation process. We answer a set of other relevant questions along the way.

A key remark is in order at this point: while the DSP model approach proposed in Figure 3 has many advantages, it is not well suited for investigating the role of the symbolic to numerical map in the identification of DNA harmonics. It follows that *a completely new paradigm for detecting DNA harmonics is required*. The main contribution of this article is therefore the derivation of a novel matrix-based framework for the computation of the DNA spectrum that is extremely well fitted to the study of the symbolic to numerical transformation. Specifically, we first derive a new matrix-based expression of the DNA spectrum that: 

1. comprises most of the existing mappings in the literature as special cases,

2. shows that the DNA spectrum is in fact invariable under all these mappings,

3. generates a necessary condition for the invariance of the DNA spectrum to the symbolic to numerical mapping used to compute it.

Furthermore, the new algebraic framework presented here decomposes the frequency identification problem into several fundamental components that are *totally independent of each other*. It follows that sophisticated digital filters and/or alternative transformations to the DFT such as the discrete cosine, sine, and Hartley transforms can *always* be easily incorporated in the harmonics detection scheme irrespective of the choice of the symbolic to numerical map. Finally, although the newly proposed framework is matrix based, we show that similar to the DSP model approach, the computation of the DNA spectrum using this new framework is very efficient.

The article is organized as follows. In Section 2, we derive a new matrix based framework to efficiently compute the ST-DFT-based spectrum. New expressions for the ST-DFT $X_{l}(\mathrm{Rn},\frac{M}{R})$ and its magnitude squared $|X_{l}(\mathrm{Rn},\frac{M}{R}){|}^{2}$ are obtained and indicate that these quantities are completely parameterized by some pre-defined matrices. The numerical values of these matrices simply depend on our choice of filtering (e.g., rectangular window versus non-rectangular one versus general FIR filters) as well as our choice of data transform (e.g., the DFT versus the DCT versus the DST).

Using these results, in Section 3, a new expression of the DNA power spectrum is derived and is also completely defined by these matrices. The elegance of this matrix based approach is that it allows the incorporation of general symbolic to numerical maps into the newly derived DNA spectrum expression *provided these generic maps can be expressed as affine transformations of the Voss representation*. This last assumption is motivated by the fact that all the popular maps that are available in the literature satisfy the affine condition. Furthermore, the maps are now completely characterized by the affine transformation (two matrices **A** and **b**) and can be therefore changed *without affecting the remaining matrices* in the DNA spectrum expression. In conclusion, the newly derived DNA spectrum expression is stated as a function of a number of matrices. Each of these matrices captures an essential component of the process (filtering, data transform, symbolic to numerical map) and the elements of each matrix can be changed without affecting the other matrices.

In Section 4 and using the above results, we show that the Voss-based DNA spectrum is essentially invariant under some of the most popular maps in the literature. A **necessary and sufficient** condition for the invariance of the DNA spectrum under any map is also derived.

In Section 5, we show how the special structure of the filtering matrix allows the efficient use of sophisticated digital filters to improve the detection performance of DNA harmonics through the computation of the DNA spectrum. We also show how to replace the DFT by other fast transforms such as the discrete cosine transform (DCT), the discrete sine transform (DST), and the discrete Hartley transform (DHT). Finally, some concluding remarks are mentioned in Section 6. A list of the different notation used in the article is summarized in Table 1.

**Table 1:** Summary of the article notations


	{*A*,*C*,*G*,*T*}, the field of DNA nucleotides
	{0,1}, the field of Voss binary elements
	A general field of complex valued elements
F↦D	Field mapping operation from set to set , resulting in *γ*sequences *x*_*l*_(*n*), where *l* = 1,…,*γ*. For example, when D=V, F↦D results in *γ* = 4 binary sequences, namely: *x*_*A*_(*n*),*x*_*C*_(*n*),*x*_*G*_(*n*), and *x*_*T*_(*n*)
*x*_*l*_(*n*)	A discrete time sequence of length *N* whose elements belong to the mapped field
**x**_*l*_(*n*)	The *n*^*th*^ window of length *M*, extracted from *x*_*l*_(*n*), *l* = 1,…,*γ*
${\hat{x}}_{l}(n)$	The interleaved version of **x**(*n*) with an interleaving factor *R*, *l* = 1,…,*γ*
$X_{l}(\mathrm{Rn},\frac{M}{R})$	The ST-DFT of *x*_*l*_(*n*), generated using a sliding window of length *M* and a window shift of length *R*
**ϒ**_*v*_(*n*)	[*X*_*A*_(*n*) *X*_*C*_(*n*) *X*_*G*_(*n*) *X*_*T*_(*n*)]^*T*^, the array of the four -based ST-DFTs
**ϒ**_*d*_(*n*)	[*X*_1_(*n*) *X*_2_(*n*) … *X*_*γ*_(*n*)]^*T*^, the array of the *γ*-based ST-DFTs
*X*_*lr*_(*n*)	The *r*^*th*^ filtered polyphase component of *X*_*l*_(*n*), where *r* = 0,1,…,*R* − 1 and *l* = 1,…,*γ*
*S*_*v*_(*n*)	The DNA spectrum computed by adding the magnitude squared of the ST-DFT of the four -based sequences
*S*_*d*_(*n*)	The DNA spectrum computed by adding the magnitude squared of the ST-DFT of the *γ*-based sequences
**Γ**_*l*_(*n*)	[*X*_*l*0_(*n*) *X*_*l*1_(*n*) … *X*_*l*,*R*−1_(*n*)]^*T*^, the array of the *R* filtered polyphase components *X*_*lr*_(*n*), *r* = 0,1,…,*R* − 1 and *l* = 1,…,*γ*
**I**_*γ*_	An identity matrix of size *γ* × *γ*
**C**	An array of length *R* whose elements are equally spaced on the unit circle
**h**	An array of length *M*/*R* whose elements are all equal to one
**D**	**C**^⋆^**C**^*T*^, an *R* × *R* matrix
**H**	**I**_*R*_ ⊗ **h**^*T*^, an *R* × *R* block matrix of $\frac{M}{R}\times 1$ blocks
**W**	**H**^*H*^**DH**, an *R* × *R* block matrix of $\frac{M}{R}\times \frac{M}{R}$ blocks
**A**,**b**	The affine transformation matrices of size *γ* × 4 and *γ* × 1, respectively, that map the four -based sequences into the *γ*-based sequences.
**B**	***A***^**H**^**A**, a 4 × 4 matrix
$\tilde{C}$	A complex valued array of *R* elements
$\tilde{h}$	A complex valued array of *M*/*R* elements
$\tilde{D}$	${\tilde{C}}^{⋆}{\tilde{C}}^{T}$, an *R* × *R* matrix
$\tilde{h}$	$I_{R}⊗{\tilde{h}}^{T}R\times R$ block matrix of $\frac{M}{R}\times 1$ blocks
$\tilde{W}$	${\tilde{H}}^{H}\tilde{D}\tilde{H}$, an *R* × *R* block matrix of $\frac{M}{R}\times \frac{M}{R}$ blocks

## 2 A new algebraic framework for computing the ST-DFT

**Figure 4:**
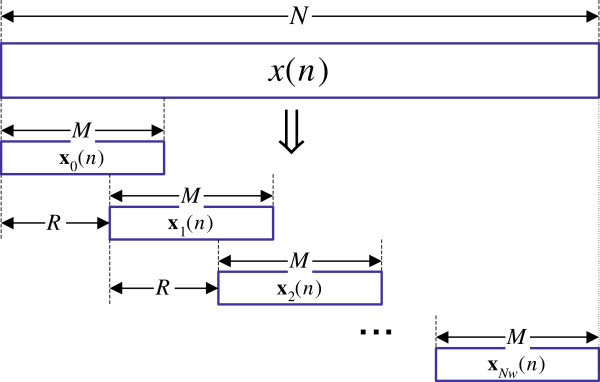
**Splitting *x*** **(*n*) into *N***_***w ***_**overlapping sections *x***_***r***_**(*n*) using a sliding window approach.**

**Figure 5:**
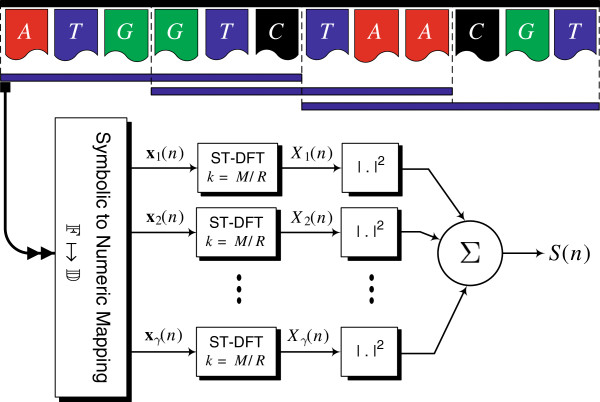
**System structure to find the DNA power spectrum *S*(*n*) by extracting successive sliding windows of the symbolic DNA sequence, mapping each to *γ *numeric sequences, finding their DFT’s at **$k=\frac{M}{R}$**, and finally adding the corresponding squared magnitudes.** In this example, *N*_*w*_ = ⌈(*N* − *M* + 1)/*R*⌉ = ⌈(12 − 6 + 1)/3⌉ = 3 windows are generated.

Given a sequence *x*(*n*) of length *N*, the ST-DFT is typically implemented using a sliding window approach as shown in Figure 4. Windows of length *M* that overlap with a factor *R* are first generated to form **x**_*r*_(*n*),*r* = 1,2,…,*N*_*w*_, where *N*_*w*_ = ⌈(*N* − *M*  + 1)/*R*⌉ is the number of resulting windows. Once we map the DNA sequence into an integer number of numeric sequences *γ*, given by *x*_*l*_(*n*), *l* = 1,…,*γ*( F↦D), the ST-DFT’s *X*_*l*_(*n*), *l* = 1,…,*γ* can be found and their squared magnitudes are added to result in the DNA Spectrum *S*(*n*) as summarized in Figure 5.

It was shown in [26] that the ST-DFT of *x*(*n*) can be written as

(3) $$\begin{array}{lcl} X(\mathrm{Rn},\frac{M}{R})=X_{0}(n)+X_{1}(n)e^{-j\frac{2\Pi }{R}}+\cdots +X_{R-1}(n)e^{-j2\Pi \frac{R-1}{R}}, & & \end{array}$$

where the quantities *X*_*r*_(*n*),∀ *r* ∈ {0,1,…,*R* − 1} are the so-called filtered polyphase sequences given by

(4) $$\begin{array}{ll} \;X_{r}(n) & ≐X_{r}\left(\mathrm{Rn},\frac{M}{R}\right)\\ & =\overset{\left\lfloor \frac{M}{R}-1\right\rfloor }{\underset{m=r,r+R,\ldots }{\sum }}x(\mathrm{Rn}+\mathrm{Rm}+r)h_{r}(m), \end{array}$$

∀ *r* ∈ {0,1,…,*R* − 1}. The impulse response *h*_*r*_(*m*) is the inverse -transform of *H*_*r*_(*z*) in Figure 3. Equations (3) and (4) can be used to compute the ST-DFT of a discrete time sequence, and subsequently its magnitude squared. In this section, we re-express these equations in matrix form, and then use the new formula to derive an expression for $|X(\mathrm{Rn},\frac{M}{R}){|}^{2}$. Throughout the article, vectors and matrices (arrays) are always expressed in bold letters. The notation for the various matrix operations is given in Table 2.

**Table 2:** Notation of matrix operations


{·}^∗^	Matrix complex conjugate
{·}^*T*^	Matrix transpose
{·}^*H*^	Matrix hermitian
{⊗}	Kronecker product of two matrices
{.}	Vector of columns of a matrix

### 2.1 Matrix formulation of the ST-DFT

Using the defined matrix notation, we can restate Equation (3) as

(5) $$\begin{array}{ll} \;X\left(\mathrm{Rn},\frac{M}{R}\right) & =\left[1\;e^{-j\frac{2\Pi }{R}}\;\cdots \;e^{-j2\Pi \frac{R-1}{R}}\right]\;\left[\begin{array}{c} X_{0}(n)\\ X_{1}(n)\\ \vdots \\ X_{R-1}(n) \end{array}\right]\\ & ≐C^{T}\Gamma (n). \end{array}$$

**Figure 6:**
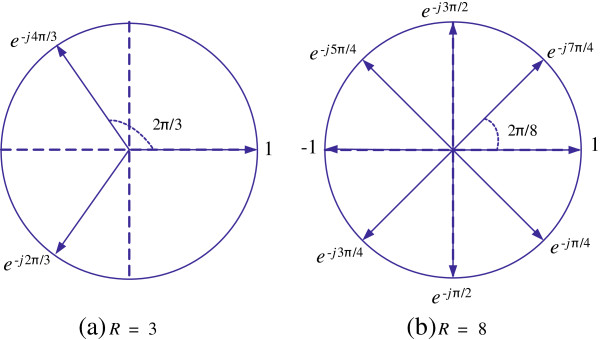
**Elements of array C of Equation (7), represented as phasors on the unit circle for (a) *R *** **= 3, and (b) *R *** **= 8.**

The real valued array

(6) $$\Gamma (n)={\left[X_{0}(n)\;X_{1}(n)\;\ldots \;X_{R-1}(n)\right]}^{T}$$

is the vector whose elements are the *R* filtered polyphase components. Similarly, the complex valued *R*-element array

(7) $$C={\left[1\;e^{-j\frac{2\Pi }{R}}\;\cdots \;e^{-j2\Pi \frac{R-1}{R}}\right]}^{T}$$

is the vector whose elements are the *R* equispaced phasors located on the unit circle with $\frac{2\Pi }{R}$ phase deviations as shown in Figure 6 for *R* = 3 and *R* = 8. Note that

(8) $$\begin{array}{lcl} \overset{R-1}{\underset{r=0}{\sum }}e^{-j2\Pi r/R}=\frac{1-{\left(e^{-j2\Pi /R}\right)}^{R}}{1-e^{-j2\Pi /R}}=0, & & \end{array}$$

∀ *R* ≠ 1, which implies that the sum of elements in **C** is equal to 0. This is a key feature of the complex array **C** that will be used in later sections to simplify important expressions.

On the other hand, we observe that (4) can be written in the following matrix format

(9) $$X_{r}(n)=\left[1\;1\;\cdots \;1\right]\;\left[\begin{array}{c} x(\mathrm{Rn}+r)\\ x(\mathrm{Rn}+r+R)\\ \vdots \\ x(\mathrm{Rn}+r+M-R) \end{array}\right]≐h^{T}{\hat{x}}_{r}(n),$$

∀ *r* ∈ {0,1,…,*R* − 1}, where **h** is an all-one vector of length *M*/*R*, and ${\hat{x}}_{r}(n)$ of length *M*/*R* is the *r*^*th*^ polyphase component of the window **x**(*n*) of length *M*. Using (9), the *R* filtered polyphase components *X*_*r*_(*n*) can be arranged in the following array format

(10) $$\begin{array}{ll} \left[X_{0}(n)\;X_{1}(n)\;\ldots \;X_{R-1}(n)\right] & =h^{T}[{\hat{x}}_{0}(n)\;{\hat{x}}_{1}(n)\;\ldots \\ & \;{\hat{x}}_{R-1}(n)]. \end{array}$$

Using the identity

(11) $$\mathrm{vec}(A_{1}A_{2})=(I⊗A_{1})\mathrm{vec}(A_{2}),$$

it follows that

$$\left[\begin{array}{c} X_{0}(n)\\ X_{1}(n)\\ \vdots \\ X_{R-1}(n) \end{array}\right]=\left(I_{R}⊗h^{T}\right)\;\left[\begin{array}{c} {\hat{x}}_{0}(n)\\ {\hat{x}}_{1}(n)\\ \vdots \\ {\hat{x}}_{R-1}(n) \end{array}\right],$$

which can be restated in matrix format as

(12) $$\begin{array}{lcl} \Gamma (n)=\left(I_{R}⊗h^{T}\right)\hat{x}(n)=H\;\hat{x}(n), & & \end{array}$$

where **H** ≐ **I**_*R*_ ⊗ **h**^*T*^ is an *R* × *R* matrix of $1\times \frac{M}{R}$ blocks, given by

$$H=\left[\begin{array}{cccc} h^{T} & 0 & \cdots & 0\\ 0 & h^{T} & \ddots & \vdots \\ \vdots & \ddots & \ddots & 0\\ 0 & \ldots & 0 & h^{T} \end{array}\right]=\left[\begin{array}{ccc} 1\;1‥1 & \cdots & 0\;0‥0\\ \vdots & \ddots & \vdots \\ 0\;0‥0 & \cdots & \underset{M/R}{\underset{︸}{1\;1‥1}} \end{array}\right].$$

The window $\hat{x}(n)$ of length *M* is a block interleaved version of the sliding window **x**(*n*) of length *M* starting at index *n*. Generating $\hat{x}(n)$ can be accomplished by blocking the window **x**(n) into an array of *R* elements per row (hence *M/R* rows), and then reading the array out column by column. The ST-DFT $X(\mathrm{Rn},\frac{M}{R})$ can therefore be completely identified as a function of **C**, **h**, and $\hat{x}(n)$ as follows

(13) $$\begin{array}{lcl} X(\mathrm{Rn},\frac{M}{R})=C^{T}\left(I_{R}⊗h^{T}\right)\hat{x}(n)=C^{T}H\;\hat{x}(n). & & \end{array}$$

The complex row vector **C**^*T*^**H** is an array of *R* blocks, each of length $\frac{M}{R}$ as given by

$$\begin{array}{l} C^{T}H=\left[\underset{M/R}{\underset{︸}{1\;..\;1}}\;\underset{M/R}{\underset{︸}{e^{-j\frac{2\Pi }{R}}\;..\;e^{-j\frac{2\Pi }{R}}}}\;\cdots \;\underset{M/R}{\underset{︸}{e^{-j2\Pi \frac{R-1}{R}}\;..\;e^{-j2\Pi \frac{R-1}{R}}}}\right], \end{array}$$

which represents *M/R* repetitions of the elements in **C**. Similar to **C**, the sum of elements in **C**^*T*^**H** is equal to 0.

### 2.2 A matrix based expression for the magnitude squared of the ST-DFT

Using (5), the magnitude squared of the ST-DFT can be expressed as

(14) $${\left|X\left(\mathrm{Rn},\frac{M}{R}\right)\right|}^{2}=X^{H}(n)X(n)≐\Gamma^{H}(n)D\Gamma (n),$$

where matrix **D** ≐ **C**^⋆^**C**^*T*^ is an *R* × *R* matrix given by

$$D=\left[\begin{array}{cccc} 1 & e^{-j\frac{2\Pi }{R}} & \cdots & e^{-j2\Pi \frac{R-1}{R}}\\ e^{j\frac{2\Pi }{R}} & 1 & \ddots & \vdots \\ \vdots & \ddots & \ddots & e^{-j\frac{2\Pi }{R}}\\ e^{j2\Pi \frac{R-1}{R}} & \cdots & e^{j\frac{2\Pi }{R}} & 1 \end{array}\right].$$

**D** is obviously a right circulant (hence Toeplitz) matrix whose rows and columns are rotated versions of **C**. Obviously, the sum of any row or column elements in **D** is equal to 0. Substituting (12) in (14), or equivalently using (13), implies that the spectrum *S*(*n*) can be stated as

(15) $$\begin{array}{ll} \;{\left|X\left(\mathrm{Rn},\frac{M}{R}\right)\right|}^{2} & ={\hat{x}}^{H}(n)\left[{\left(I_{R}⊗h^{T}\right)}^{H}C^{⋆}\right]\\ & \;\times \left[C^{T}\left(I_{R}⊗h^{T}\right)\right]\hat{x}(n)\\ & ={\hat{x}}^{H}(n)\;H^{H}D\;H\;\hat{x}(n)\\ & ={\hat{x}}^{H}(n)\;W\;\hat{x}(n), \end{array}$$

where

$$\begin{array}{lcl} W≐H^{H}DH={\left(C^{T}H\right)}^{H}(C^{T}H), & & \end{array}$$

is an *R* × *R* matrix of $\frac{M}{R}\times \frac{M}{R}$ blocks, given by

$$W=\left[\begin{array}{cccc} 1 & e^{-j\frac{2\Pi }{R}} & \cdots & e^{-j2\Pi \frac{R-1}{R}}\\ e^{j\frac{2\Pi }{R}} & 1 & \ddots & \vdots \\ \vdots & \ddots & \ddots & e^{-j\frac{2\Pi }{R}}\\ \underset{\frac{M}{R}\times \frac{M}{R}}{\underset{︸}{e^{j2\Pi \frac{R-1}{R}}}} & \ldots & e^{j\frac{2\Pi }{R}} & \underset{\frac{M}{R}\times \frac{M}{R}}{\underset{︸}{1}} \end{array}\right].$$

Matrix **W** can be represented as a Kronecker product of **D** and an $\frac{M}{R}\times \frac{M}{R}$ all-one matrix. Note that any row or column in **W** is a rotated version of **C**^*T*^**H**, therefore, the sum of the elements of any row or column in **W** is equal to 0.

## 3 The new DNA spectrum expression

A first step towards finding the DNA spectrum *S*(*n*) is the symbolic to numeric mapping F↦D as was shown in Figure 5. Once the symbolic DNA sequence is mapped into *γ*numeric sequence(s), the short-time discrete Fourier transform is applied to each of them and the sum of the squared magnitudes of the ST-DFTs will result in the DNA spectrum at the frequency point $k=\frac{M}{R}$ as given by

(16) $$\begin{array}{lcl} S(\mathrm{Rn},k){|}_{k=\frac{M}{R}}=\overset{\gamma }{\underset{l=1}{\sum }}{\left|X_{l}\left(\mathrm{Rn},\frac{M}{R}\right)\right|}^{2}. & & \end{array}$$

For simplicity, we denote $S(\mathrm{Rn},k){|}_{k=\frac{M}{R}}$ as *S*(*n*) in the following sections. Several mappings were introduced in the literature using both real and complex numerical values with typical number of sequences *γ* = 1 up to 4 to maintain reasonable computation complexity. In this section, we use the results of Section 2 to derive general expressions for the *M/R* ST-DFT and spectrum for any symbolic to numeric mapping.

### 3.1 The Voss-based DNA spectrum

The simplest and most commonly used map of a DNA sequence is the Voss representation F↦V: that is to form *γ* = 4 binary indicator sequences *x*_*A*_(*n*),*x*_*C*_(*n*), *x*_*G*_(*n*), and *x*_*T*_(*n*) where a 1 would indicate the presence of a base and 0 indicates its absence [18]. This approach has been extensively used in relevant genomic research. Note that the four sequences are not linearly independent since for any index *n*, the four sequences will add up to one. That is

$$\begin{array}{lcl} x_{A}(n)+x_{C}(n)+x_{G}(n)+x_{T}(n)=1. & & \end{array}$$

This redundancy plays an important role in the derivations of this section. Moreover, it follows that for any length-*M* window starting at *n*, the four mapped Voss windows will add up to an all-one length-*M* sequence and the same fact holds for the interleaved windows

(17) $$\begin{array}{ll} \;x_{A}(n)+x_{C}(n)+x_{G}(n)+x_{T}(n) & ={\hat{x}}_{A}(n)+{\hat{x}}_{C}(n)\\ & \;+{\hat{x}}_{G}(n)+{\hat{x}}_{T}(n)\\ & ={\left[1\;1\;\cdots \;1\right]}^{T}. \end{array}$$

**Figure 7:**
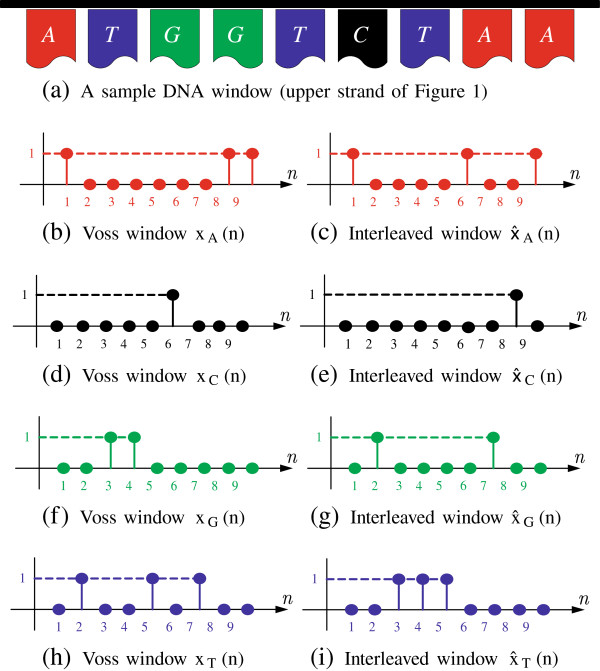
**A sample DNA window of length *M *= 9, the corresponding Voss binary windows **$x_{l}(n),\forall l\in F$**, and the interleaved versions **${\hat{x}}_{l}(n),\forall l\in F$**with an interleaving factor *R *= 3.** The interleaved windows are generated by rearranging the original windows in an *R* = 3-interleaved format. In this example, data points of ${\hat{x}}_{l}(n)$ at (1,2,3),(4,5,6),(7,8,9) are mapped from those in **x**_*l*_(*n*) at (1,4,7),(2,5,8),(3,6,9).

For illustration, Figure 7a shows a sample DNA window that is mapped into the corresponding numeric windows $x_{l}(n),\forall \;l\in F$ in Figure 7b,d,f,h. With an example interleaving factor *R* = 3, the interleaved windows ${\hat{x}}_{l}(n),\forall \;l\in F$ are shown in Figure 7c,e,g,i. Each of the four sequences is a discrete time sequence that can be processed using the analysis of Section 2.

Therefore, the ST-DFT of each sequence can be found using (13) to be

(18) $$\begin{array}{c} X_{l}(n)=C^{T}H\;{\hat{x}}_{l}(n), \end{array}$$

∀l∈F, and the power spectrum of each sequence can hence be derived as in (16) to be

$$\begin{array}{c} S_{l}(n)=|X_{l}(n){|}^{2}={\hat{x}}_{l}^{H}(n)\;W\;{\hat{x}}_{l}(n), \end{array}$$

∀l∈F. It follows that the Voss-based DNA spectrum *S*_*v*_(*n*) is

(19) $$\begin{array}{ll} \;S_{v}(n) & ≐|X_{A}(n){|}^{2}+|X_{C}(n){|}^{2}+|X_{G}(n){|}^{2}+|X_{T}(n){|}^{2}\\ & =\underset{l\in F}{\sum }{\hat{x}}_{l}^{H}(n)\;W\;{\hat{x}}_{l}(n). \end{array}$$

An obvious step at this point is to simplify (19) to avoid the summation over different bases. To do this, we use Equation (18) to arrange the ST-DFT’s of *x*_*l*_(*n*), ∀l∈F in the following format

(20) $$\begin{array}{ll} \;[X_{A}(n)X_{C}(n)X_{G}(n)X_{T}(n)] & =C^{T}H\;[{\hat{x}}_{A}(n)\;{\hat{x}}_{C}(n)\\ & \;{\hat{x}}_{G}(n)\;{\hat{x}}_{T}(n)]. \end{array}$$

Using (11), it follows that

$$\begin{array}{l} \left[\begin{array}{c} X_{A}(n)\\ X_{C}(n)\\ X_{G}(n)\\ X_{T}(n) \end{array}\right]=\left(\left[\begin{array}{cccc} 1 & 0 & 0 & 0\\ 0 & 1 & 0 & 0\\ 0 & 0 & 1 & 0\\ 0 & 0 & 0 & 1 \end{array}\right]⊗C^{T}H\right).\left[\begin{array}{c} {\hat{x}}_{A}(n)\\ {\hat{x}}_{C}(n)\\ {\hat{x}}_{G}(n)\\ {\hat{x}}_{T}(n) \end{array}\right]. \end{array}$$

We define **ϒ**_*v*_(*n*): the array of the four Voss-based ST-DFTs. It can now be written as

(21) $$\begin{array}{ll} \;{ϒ}_{v}(n) & ={\left[X_{A}(n)X_{C}(n)X_{G}(n)X_{T}(n)\right]}^{T}\\ & =\left(I_{4}⊗C^{T}H\right){\hat{x}}_{v}(n), \end{array}$$

where **I**_4_ is the 4 × 4 identity matrix, and the vector ${\hat{x}}_{v}(n)$ of length 4*M* is an array of the four Voss interleaved windows starting at index *n*: ${\hat{x}}_{l}(n),\forall \;l\in F$. Using the identity

(22) $$(A_{1}⊗A_{2})(A_{3}⊗A_{4})=(A_{1}A_{3}⊗A_{2}A_{4}),$$

the Voss-based DNA power spectrum can be manipulated into

(23) $$\begin{array}{ll} \;S_{v}(n) & ≐{ϒ}_{v}^{H}(n){ϒ}_{v}(n)\\ & ={\hat{x}}_{v}^{H}(n)\left(I_{4}^{H}⊗{\left(C^{T}H\right)}^{H}\right)\left(I_{4}⊗C^{T}H\right){\hat{x}}_{v}(n)\\ & ={\hat{x}}_{v}^{H}(n)\left(I_{4}⊗W\right){\hat{x}}_{v}(n). \end{array}$$

In (26), **I**_4_ and **W** are constant matrices ∀*n*. Hence the computation of the spectrum *S*_*v*_(*n*) for different windows of a DNA sequence needs only the evaluation of the Voss interleaved array ${\hat{x}}_{v}(n)$.

### 3.2 Computing the DNA spectrum under general symbolic to numerical maps

Similar to the Voss representation case, any map F↦D of *γ*sequences can be processed using the analysis of Section 2. It directly follows that the ST-DFT and spectrum of a single sequence are given by

$$\begin{array}{l} X_{l}(n)=C^{T}H\;{\hat{x}}_{l}(n),\\ S_{l}(n)={\hat{x}}_{l}^{H}(n)\;W\;{\hat{x}}_{l}(n), \end{array}$$

where *l* = 1,2,…,*γ*. The array of γD-mapped ST-DFTs **ϒ**_*d*_(*n*) is therefore given by

(24) $$\begin{array}{ll} \;{ϒ}_{d}(n) & ={\left[X_{1}(n)X_{2}(n)\ldots X_{\gamma }(n)\right]}^{T}\\ & =\left(I_{\gamma }⊗C^{T}H\right){\hat{x}}_{d}(n). \end{array}$$

The -based DNA spectrum can easily be shown to be

(25) $$S_{d}(n)={\hat{x}}_{d}^{H}(n)\left(I_{\gamma }⊗W\right){\hat{x}}_{d}(n),$$

where the vector ${\hat{x}}_{d}(n)$ of length *γM* is an array of the *γ*-mapped and interleaved windows starting at index *n*: ${\hat{x}}_{l}(n),\forall \;l=1,2,\ldots ,\gamma$. It is clear that for every different map F↦D, a new interleaved windows array ${\hat{x}}_{d}(n)$ has to be evaluated in order to compute a spectrum point *S*_*d*_(*n*). In this following, we introduce a different new approach to recompute (28) without updating ${\hat{x}}_{d}(n)$ for every map. Basically, we derive a new expression for *S*_*d*_(*n*) in terms of ${\hat{x}}_{v}(n)$ and a new constant matrix so that we incorporate the map dependance in the matrix part rather than the interleaved array part. In other words, since the map F↦V is already well-defined, we use the map V↦D to complete the chain F↦V↦D and hence find the spectrum *S*_*d*_(*n*). Consider the following affine transformation from Voss sequences to a general array of -mapped sequences

$$\begin{array}{l} {\left[\begin{array}{c} x_{1}(n)\\ x_{2}(n)\\ \vdots \\ x_{\gamma }(n) \end{array}\right]}_{\gamma \times 1}=A_{\gamma \times 4}{\left[\begin{array}{c} x_{A}(n)\\ x_{C}(n)\\ x_{G}(n)\\ x_{T}(n) \end{array}\right]}_{4\times 1}+b_{\gamma \times 1}, \end{array}$$

where **A**_*γ*×4_ and **b**_*γ*×1_ = [*b*_1_*b*_2_ … *b*_*γ*_]^*T*^ are constant possibly complex valued arrays. It follows that the array of the -mapped interleaved windows ${\hat{x}}_{d}(n)$ can be written in terms of the array the Voss-mapped interleaved windows ${\hat{x}}_{v}(n)$ in the following form

(26) $${\hat{x}}_{d}{\left(n\right)}_{\gamma M\times 1}=\left(A_{\gamma \times 4}⊗I_{M}\right){\hat{x}}_{v}{\left(n\right)}_{4M\times 1}+{\hat{b}}_{\gamma M\times 1},$$

where $\hat{b}$ defined as

$$\hat{b}=\left[\underset{M}{\underset{︸}{b_{1}..b_{1}}}\;b_{2}\;..\;b_{2}\ldots \underset{M}{\underset{︸}{b_{\gamma }\;..\;b_{\gamma }}}\right]$$

is an array of *γM*-element blocks, each block is *M* repetitions of one element of **b**. Substituting for ${\hat{x}}_{d}(n)$ in (24) results in a new formula for the array of -mapped ST-DFTs **ϒ**_*d*_(*n*) into

(27) $${ϒ}_{d}(n)=\left(I_{\gamma }⊗C^{T}H\right)\left[\left(A⊗I_{M}\right){\hat{x}}_{v}(n)+\hat{b}\right].$$

An important result at this point is that the second term in **ϒ**_*d*_(*n*) is actually equal to 0. This can be verified by reducing it into the following form

$$\left(I_{\gamma }⊗C^{T}H\right)\hat{b}=\left[\begin{array}{cccc} C^{T}H & 0 & \cdots & 0\\ 0 & C^{T}H & \ddots & \vdots \\ \vdots & \ddots & \ddots & 0\\ 0 & \ldots & 0 & C^{T}H \end{array}\right]\;\left[\begin{array}{c} {\hat{b}}_{1}\\ {\hat{b}}_{2}\\ \vdots \\ {\hat{b}}_{\gamma } \end{array}\right].$$

Recall that the sum of elements in **C**^*T*^**H** is equal to 0. Therefore, since ${\hat{b}}_{l}$ is a constant vector, the product $\left(C^{T}H\right).{\hat{b}}_{l}$ is equal to 0, ∀ *l* = 1,2,…,*γ* and hence

(28) $$\left(I_{\gamma }⊗C^{T}H\right)\hat{b}=\overset{\gamma }{\underset{l=1}{\sum }}\left(C^{T}H\right).{\hat{b}}_{l}=0.$$

The ST-DFTs array **ϒ**_*d*_(*n*) can therefore be simplified using the Kronecker product identity (22) into

(29) $$\begin{array}{ll} \;{ϒ}_{d}(n) & =\left(I_{\gamma }⊗C^{T}H\right)\left(A⊗I_{M}\right){\hat{x}}_{v}(n)\\ & =\left(A⊗C^{T}H\right){\hat{x}}_{v}(n). \end{array}$$

It follows that the -based DNA spectrum *S*_*d*_(*n*) is

(30) $$\begin{array}{ll} \;S_{d}(n) & ={ϒ}_{d}^{H}(n){ϒ}_{d}(n)\\ & ={\hat{x}}_{v}^{H}(n){\left(A⊗C^{T}H\right)}^{H}\left(A⊗C^{T}H\right){\hat{x}}_{v}(n)\\ & ={\hat{x}}_{v}^{H}(n)\left(B⊗W\right){\hat{x}}_{v}(n), \end{array}$$

where **B** ≐ **A**^*H*^**A**. Equation (35) indicates that when a certain symbolic to numeric mapping F↦D is used, the DNA power spectrum *S*_*d*_(*n*) is completely defined in terms of the Voss-based interleaved array ${\hat{x}}_{v}(n)$ along with constant matrices **W** and **B** which is a function of the transformation matrix **A** ( V↦D). Note that if **A** = **I**_4_ then **B** = **I**_4_ at which (35) reduces to (26) which is the Voss-based spectrum case.

## 4 Invariance of the DNA spectrum under popular mappings

The results found in Section 3 can be applied to some mappings that are widely used in the literature. In specific, by defining the corresponding transformation matrices **A** and **B** ( V↦D), closed form expressions for *S*_*d*_(*n*) are obtained. Furthermore, for a number of mappings, we show that the -mapped spectrum *S*_*d*_(*n*) is in fact a scaled version of the Voss-based spectrum *S*_*v*_(*n*).

### 4.1 Four-to-four (*γ* = 4) representations

In this scheme, each Voss sequence is scaled by a possibly complex coefficient according to the following transformations matrices

$$A=\left[\begin{array}{cccc} a & 0 & 0 & 0\\ 0 & c & 0 & 0\\ 0 & 0 & g & 0\\ 0 & 0 & 0 & t \end{array}\right],\;B=\left[\begin{array}{cccc} |a{|}^{2} & 0 & 0 & 0\\ 0 & |c{|}^{2} & 0 & 0\\ 0 & 0 & |g{|}^{2} & 0\\ 0 & 0 & 0 & |t{|}^{2} \end{array}\right],$$

where *a*, *a*, *g*, and *t* are real or complex coefficients used to scale *x*_*A*_(*n*),*x*_*C*_(*n*),*x*_*G*_(*n*), and *x*_*T*_(*n*), respectively. The corresponding array of ST-DFT’s **ϒ**_*d*_(*n*) is subsequently given by

$${ϒ}_{d}(n)=\left(\left[\begin{array}{cccc} a & 0 & 0 & 0\\ 0 & c & 0 & 0\\ 0 & 0 & g & 0\\ 0 & 0 & 0 & t \end{array}\right]⊗C^{T}H\right)\;{\hat{x}}_{v}(n),$$

and the DNA spectrum *S*_*d*_(*n*) is

$$S_{d}(n)={\hat{x}}_{v}^{H}(n)\left(\left[\begin{array}{cccc} |a{|}^{2} & 0 & 0 & 0\\ 0 & |c{|}^{2} & 0 & 0\\ 0 & 0 & |g{|}^{2} & 0\\ 0 & 0 & 0 & |t{|}^{2} \end{array}\right]⊗W\right)\;{\hat{x}}_{v}(n).$$

Now, we extend this result to certain transformations where numeric values of the scale factors *a*, *a*, *g*, and *t* are specified.

§ *Tetrahedral mapping.*

The so-called tetrahedral representation has been proposed in [13,29]. In this mapping scheme, the four nucleotides are represented by four equal length vectors oriented towards the corners of a tetrahedron. Projecting the basic tetrahedron on a plane will reduce the dimensionality of the representation to two. This mapping can be defined by the mapping matrix

$$A=\left[\begin{array}{cccc} 1+j & 0 & 0 & 0\\ 0 & -1+j & 0 & 0\\ 0 & 0 & -1-j & 0\\ 0 & 0 & 0 & 1-j \end{array}\right].$$

It can be easily seen that in this case: $|a|=|c|=|g|=|t|=\sqrt{2}$ which implies that **B** = 2**I**_**4**_. The corresponding DNA spectrum is

(31) $$S_{d}(n)=2{\hat{x}}_{v}^{H}(n)\left(I_{4}⊗W\right){\hat{x}}_{v}(n)=2S_{v}(n).$$

Since **B** = *α***I**_4_(*α* = 2), the tetrahedral-based DNA spectrum is a scaled version of the Voss-based spectrum.

§ *Quaternion mapping.*

A more involved step is to replace the complex number set of the tetrahedral mapping with its algebraic generalization, the set of quaternions. Quaternions have been used to map DNA sequences F↦H[30] and are simply defined as hypercomplex numbers given by p∈H={a+bi+cj+dk|a,b,c,d∈R}, where *i,j,k* are complex coefficients such that *i*^2^ = *j*^2^ = *k*^2^ = *ijk* = −1 and $|p|=\sqrt{pp^{*}}=\sqrt{a^{2}+b^{2}+c^{2}+d^{2}}$. The transformation matrix is given by

$$\begin{array}{c} A=\left[\begin{array}{cccc} i+j+k & 0 & 0 & 0\\ 0 & i-j-k & 0 & 0\\ 0 & 0 & -i-j+k & 0\\ 0 & 0 & 0 & -i+j-k \end{array}\right]. \end{array}$$

In this case, $|a|=|c|=|g|=|t|=\sqrt{3},B=3I_{4}$. The corresponding DNA spectrum is

(32) $$\begin{array}{c} S_{d}(n)=3{\hat{x}}_{v}^{H}(n)\left(I_{4}⊗W\right){\hat{x}}_{v}(n)=3S_{v}(n) \end{array}$$

§ *Higher order mappings.*

An alternative Quaternion transformation is given by **A** = diag(1 + *i* + *j* + *k*,1 + *i* − *j* − *k*,1 − *i* − *j* + *k*,1 − *i* + *j* − *k*), which results in **B** = 4**I**_**4**_ and consequently *S*_*d*_(*n*) = 4*S*_*v*_(*n*). In general, for a complex representation system with *η* dimensions and equal amplitude coefficients: **B** = *η***I**_**4**_ and hence the spectrum *S*_*d*_(*n*) = *ηS*_*v*_(*n*).

### 4.2 Four-to-three (*γ* = 3) mappings

In order to reduce the DNA spectrum computational cost, several mappings have been proposed with smaller numbers of sequences.

§ *-curve mapping.*

One such important symbolic-to-numeric map is the -curve mapping [24], which is a unique 3-dimensional curve representation whose sequences have values 1 and −1. One advantage of the -curve mapping is that each of its three sequences has a biological interpretation. This scheme is given by

$$\begin{array}{c} \left[\begin{array}{c} x(n)\\ y(n)\\ z(n) \end{array}\right]=2\left[\begin{array}{cccc} 1 & 0 & 1 & 0\\ 1 & 1 & 0 & 0\\ 1 & 0 & 0 & 1 \end{array}\right]\;\left[\begin{array}{c} x_{A}(n)\\ x_{C}(n)\\ x_{G}(n)\\ x_{T}(n) \end{array}\right]-\left[\begin{array}{c} 1\\ 1\\ 1 \end{array}\right]. \end{array}$$

Therefore, the transformation matrices are

$$\begin{array}{c} A=\left[\begin{array}{cccc} 2 & 0 & 2 & 0\\ 2 & 2 & 0 & 0\\ 2 & 0 & 0 & 2 \end{array}\right],\;B=\left[\begin{array}{cccc} 12 & 4 & 4 & 4\\ 4 & 4 & 0 & 0\\ 4 & 0 & 4 & 0\\ 4 & 0 & 0 & 4 \end{array}\right]. \end{array}$$

Matrix **B** in this case can be written as

$$\begin{array}{ll} \;B & =4\left(I_{4}+\left[\begin{array}{cccc} 1 & 1 & 1 & 1\\ 0 & 0 & 0 & 0\\ 0 & 0 & 0 & 0\\ 0 & 0 & 0 & 0 \end{array}\right]+\left[\begin{array}{cccc} 1 & 0 & 0 & 0\\ 1 & 0 & 0 & 0\\ 1 & 0 & 0 & 0\\ 1 & 0 & 0 & 0 \end{array}\right]\right)\\ & =4\left(I_{4}+B_{1}+B_{2}\right). \end{array}$$

Note that the term involving **B**_1_ in *S*_*d*_(*n*) can be manipulated into

$$\begin{array}{ll} \;S_{d}(n){|}_{B_{1}} & ={\hat{x}}_{v}^{H}(n)\left(B_{1}⊗W\right){\hat{x}}_{v}(n)\\ & =4{\hat{x}}_{v}^{H}(n)\left[\begin{array}{cccc} W & W & W & W\\ 0 & 0 & 0 & 0\\ 0 & 0 & 0 & 0\\ 0 & 0 & 0 & 0 \end{array}\right]\;\left[\begin{array}{c} {\hat{x}}_{A}(n)\\ {\hat{x}}_{C}(n)\\ {\hat{x}}_{G}(n)\\ {\hat{x}}_{T}(n) \end{array}\right]\;\\ & =4{\hat{x}}_{v}^{H}(n)\left[\;\begin{array}{c} W\left(\sum_{l\in F}{\hat{x}}_{l}(n)\right)\\ 0\\ 0\\ 0 \end{array}\right]. \end{array}$$

Recall from (19) that $\sum_{l\in F}{\hat{x}}_{l}(n)={\left[1\;1\cdots 1\right]}^{T}$. Take also into consideration that the sum of elements of any row or column in **W** is equal to 0. This implies that $W\left(\sum_{l\in F}{\hat{x}}_{l}(n)\right)=0$, at which it is easy to see that $S_{d}(n){|}_{B_{1}}=0$. Similarly, $S_{d}(n){|}_{B_{2}}=0$. Therefore, only the first term in **B** contributed to *S*_*d*_(*n*) at which the -curve mapped DNA spectrum is a scaled version of the Voss-based DNA spectrum

(33) $$\begin{array}{c} S_{d}(n)={\hat{x}}_{v}^{H}(n)\left(4I_{4}⊗W\right){\hat{x}}_{v}(n)=4S_{v}(n). \end{array}$$

This ratio is consistent with the result we first derived in [24] for *R* = 3, but is now shown to be general for any value of *R*. We are now ready to state an important result.

**Theorem.** *Necessary and Sufficient condition for the invariance of the DNA spectrum.* Consider the following affine transformation from Voss sequences to a general array of -mapped sequences

$$\begin{array}{c} {\left[\begin{array}{c} x_{1}(n)\\ x_{2}(n)\\ \vdots \\ x_{\gamma }(n) \end{array}\right]}_{\gamma \times 1}=A_{\gamma \times 4}{\left[\begin{array}{c} x_{A}(n)\\ x_{C}(n)\\ x_{G}(n)\\ x_{T}(n) \end{array}\right]}_{4\times 1}+b_{\gamma \times 1}, \end{array}$$

where **A**_*γ*×4_ and **b**_*γ*×1_ = [*b*_1_*b*_2_ … *b*_*γ*_]^*T*^ are constant possibly complex valued arrays. Define the 4 × 4 matrix **B** = **A**^**H**^**A**. The DNA spectrum is invariant under this map, i.e., *S*_*d*_(*n*) = *αS*_*v*_(*n*) if the transformation matrix **B** can be written as $B=\alpha I_{4}+\sum_{i}B_{i}$, where **B**_*i*_ holds constant rows and/or constant columns ∀ *i*.

The proof follows by simply observing that if **B**_*i*_ has constant rows and/or constant columns, then $S_{d}(n){|}_{B_{i}}=0$. We remind the reader at this point that the vector **b**_*γ*×1_ has no bearing on the invariance of the DNA spectrum.

§ *Simplex mapping.*

The simplex mapping is essentially another tetrahedron structured mapping that aims to eliminate the computational redundancy. Its transformations matrices are

$$\begin{array}{c} A=\frac{1}{3}\left[\begin{array}{cccc} 0 & -\sqrt{2} & -\sqrt{2} & 2\sqrt{2}\\ 0 & \sqrt{6} & -\sqrt{6} & 0\\ 3 & -1 & -1 & -1 \end{array}\right],\;B=\frac{1}{3}\left[\begin{array}{cccc} 1 & -1 & -1 & -1\\ -1 & 1 & -1 & -1\\ -1 & -1 & 1 & -1\\ -1 & -1 & -1 & 1 \end{array}\right]. \end{array}$$

Matrix **B** in this case can be written as

$$\begin{array}{c} B=\left(\frac{4}{3}\right)\;\left(I_{4}-\frac{1}{4}\left[\begin{array}{cccc} 1 & 1 & 1 & 1\\ 1 & 1 & 1 & 1\\ 1 & 1 & 1 & 1\\ 1 & 1 & 1 & 1 \end{array}\right]\right)=\left(\frac{4}{3}\right)\;\left(I_{4}+B_{1}\right). \end{array}$$

Similar to the -curve case, $S_{d}(n){|}_{B_{1}}=0$. It follows that the simplex-based DNA spectrum is also a scaled version of the Voss-based spectrum, and is given by

(34) $$\begin{array}{c} S_{d}(n)={\hat{x}}_{v}^{H}(n)\left(\frac{4}{3}I_{4}⊗W\right){\hat{x}}_{v}(n)=\left(\frac{4}{3}\right)S_{v}(n). \end{array}$$

This ratio is consistent with the result in [31] which was limited to direct DFT and is now shown to be extended to *M*/*R* ST-DFT with any value of *R*.

### 4.3 Four-to-two (*γ* = 2) mappings

Pairing couples of nucleotides together was proposed in the literature in order to exploit certain biological features in addition to complexity reduction. For example, it was suggested that exons are rich in nucleotides *C* and *G*, while introns have more *A* and *T*[29]. This claim inspired the transformation

$$\begin{array}{c} A=\left[\begin{array}{cccc} 0 & 1 & 1 & 0\\ -1 & 0 & 0 & -1 \end{array}\right],\;B=\left[\begin{array}{cccc} 1 & 0 & 0 & 1\\ 0 & 1 & 1 & 0\\ 0 & 1 & 1 & 0\\ 1 & 0 & 0 & 1 \end{array}\right]. \end{array}$$

It is obvious that the DNA spectrum in this case can be simplified to

(35) $$\begin{array}{c} S_{d}(n)={\hat{x}}_{v}^{H}(n)\;\left[\begin{array}{cccc} W & 0 & 0 & W\\ 0 & W & W & 0\\ 0 & W & W & 0\\ W & 0 & 0 & W \end{array}\right]\;{\hat{x}}_{v}(n), \end{array}$$

which obviously is not a scaled version of *S*_*v*_(*n*) since **B** in this case can not be written as $\alpha I_{4}+\sum_{i}B_{i}$, where **B**_*i*_ holds constant rows and/or constant columns ∀ *i*.

### 4.4 Four-to-one (*γ* = 1) mappings

Single sequence representations can be generated by assigning each nucleotide a certain coefficient [4,13] in order to keep the single sequence structure using the transformation array and matrix

$$\begin{array}{c} A=\left[\begin{array}{cccc} a & c & g & t \end{array}\right],\;B=\left[\begin{array}{cccc} |a{|}^{2} & a^{*}c & a^{*}g & a^{*}t\\ c^{*}a & |c{|}^{2} & c^{*}g & c^{*}t\\ g^{*}a & g^{*}c & |g{|}^{2} & g^{*}t\\ t^{*}a & t^{*}c & t^{*}g & |t{|}^{2} \end{array}\right]. \end{array}$$

Note that the coefficients chosen for the tetrahedral, quaternion, and paired coupled mappings can be reused along with the single sequence formulation. For example, the paired couples case can be reformulated in a single sequence of 1’s and −1’s using $A=\left[-1\;1\;1-1\right]$ and

$$\begin{array}{c} B=\left[\begin{array}{cccc} 1 & -1 & -1 & 1\\ -1 & 1 & 1 & -1\\ -1 & 1 & 1 & -1\\ 1 & -1 & -1 & 1 \end{array}\right], \end{array}$$

at which the DNA spectrum is

$$\begin{array}{c} S_{d}(n)={\hat{x}}_{v}^{H}(n)\;\left[\begin{array}{cccc} W & -W & -W & W\\ -W & W & W & -W\\ -W & W & W & -W\\ W & -W & -W & W \end{array}\right]\;{\hat{x}}_{v}(n). \end{array}$$

Similar to the previous case, *S*_*d*_(*n*) is not a scaled version of *S*_*v*_(*n*).

#### Experimental verification

**Figure 8:**
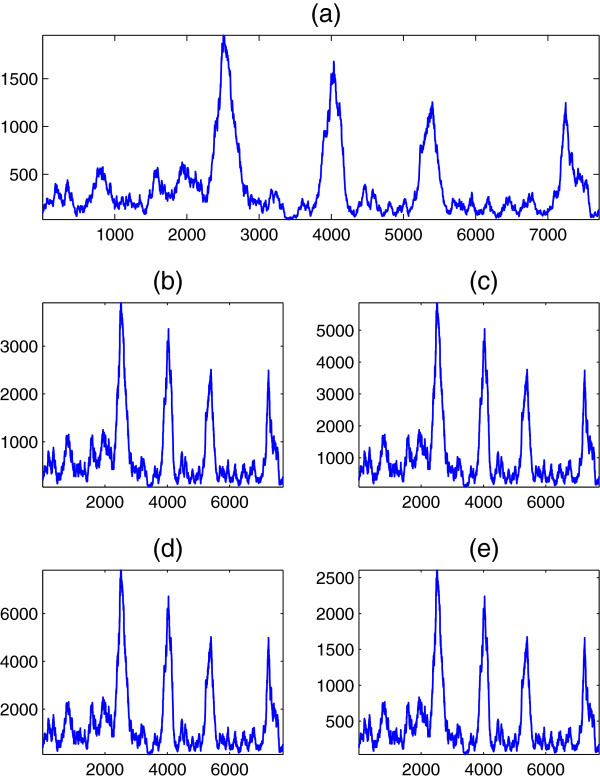
**DNA spectrum *S***_***d***_**(*n*) at *R *** **= 3 of the DNA sequence F56F11.4 when (a) Voss, (b) tetrahedral, (c) quaternion, (d) *Z*-curve, and (e) simplex mappings are used.**

To briefly verify the results of this section experimentally, we apply Equation (35) to real DNA sequences, when the Voss, tetrahedral, quaternion, Z-curve, and simplex maps are employed. For comparison with previous study, we consider first the DNA sequence F56F11.4 in the *C*. *elegans* chromosome III. This sequence is 8060 nucleotides and has been used as a benchmark by many researchers [13] to extract the periodicity component at *R* = 3. The DNA spectra at *R* = 3 are shown in Figure 8 for the five former mappings, and are obviously related by the constant scale factors derived earlier in the section which clearly verifies our results. Although we lack the space for more general simulations, it is important to state that all the spectra relations are maintained experimentally at other values of *R* associated with higher order periodicities.

**Figure 9:**
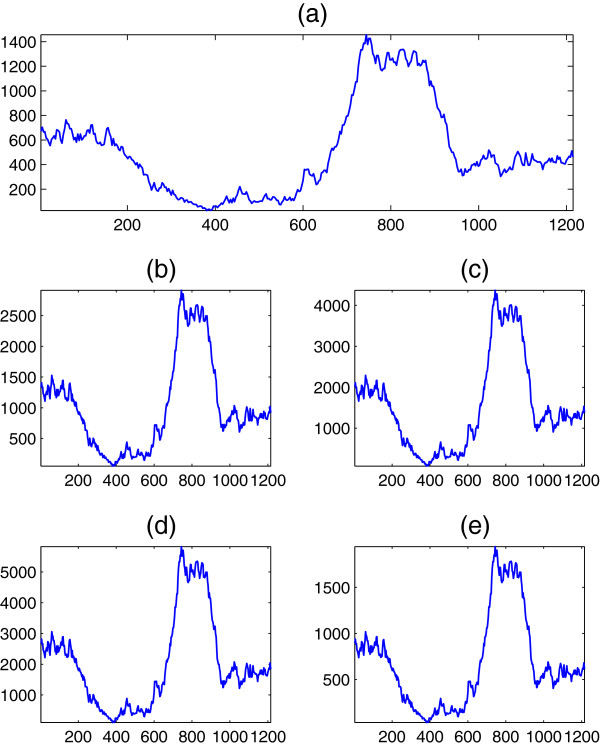
**DNA spectrum *S***_***d***_**(*n*) at *R *** **= 3 of the DNA sequence ECZGL2 when (a) Voss, (b) tetrahedral, (c) quaternion, (d) *Z*-curve, and (e) simplex mappings are used.**

**Figure 10:**
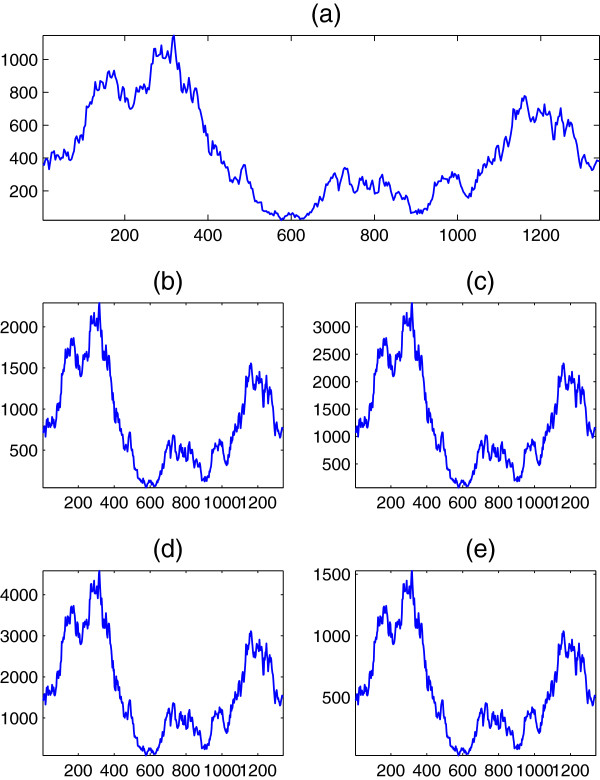
**DNA spectrum *S***_***d***_**(*n*) at *R *** **= 3 of the DNA sequence ALOEGLOBIM when (a) Voss, (b) tetrahedral, (c) quaternion, (d) *Z*-curve, and (e) simplex mappings are used.**

For generality purposes, we test two more sequences extracted from the well known Burset-Guigo database [32]. In specific, DNA spectra at *R* = 3 of the zeta globin gene (ECZGL2) of length 1563, and the Alouatta seniculus epsilon-globin gene (ALOEGLOBIM) of length 1691 are shown in Figures 9 and 10, respectively, for the five former mappings. It can be seen that the relations are still preserved.

## 5 Alternative measures of DNA periodicities

Alternative DNA periodicity measures using fast data transforms [33-35], wavelets, and finite impulse response (FIR) digital filters [25,36] were recently proposed to improve the detection performance of these periodicities. However, each method was obtained separately from the other using seemingly a different approach. In this section, we show that our proposed framework can systematically generate all these results by simply changing a number of matrices. It therefore provides a *generic unified framework* for generating alternative measures of DNA periodicities. For example, we can re-express the matrices **D** and **W** in terms of general digital filters and use these filters to modify (35) in order to generate new spectrum formulas. Furthermore, using symmetry based decompositions of **D** and **W**, we simplify (35) into a formula with low computational complexity.

### 5.1 Modified periodicity measures

Recall from Section ‘2’ that matrix **W** is given by

$$\begin{array}{c} W=H^{H}DH={\left(I_{R}⊗h^{T}\right)}^{H}C^{⋆}C^{T}(I_{R}⊗h^{T}). \end{array}$$

Obviously, **W** is completely defined by the real array **h** and the generally complex array **C**. Note that **h** and **C** can be viewed as the impulse responses of two FIR filters defined by the *z*-transforms *H*(*z*) and *C*(*z*).

#### 5.1.1 Updating the real filter **h**

The FIR filter *H*(*z*) is the standard rectangular window filter and has a low pass frequency response with a −13 dB attenuation. To improve its filtering performance, we can use a more general FIR filter, denoted by $\tilde{H}(z)$ and expressed as

$$\begin{array}{c} \tilde{H}(z)=h_{0}+h_{1}z^{-1}+\cdots +h_{\frac{M}{R}}z^{-\frac{M}{R}}, \end{array}$$

which is the -transform of the general array $\tilde{h}$ given by

$$\begin{array}{c} \tilde{h}=\left[h_{0}\;h_{1}\;\ldots \;h_{\frac{M}{R}}\right]. \end{array}$$

**Figure 11:**
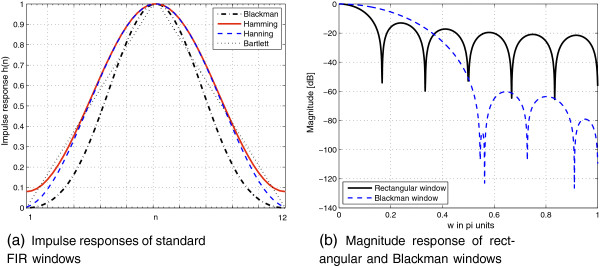
**Comparison between standard FIR windows showing (a) impulse response, (b) magnitude response, when *R *** **= 8 and *M *** **= 96.**

From a signal processing perspective, achieving better performance can be obtained by replacing the rectangular window with another one, $\tilde{H}(z)$, that has slightly wider main lobes but much more attenuated side lobes, as shown in Table 3. The impulse responses of such windows are depicted in Figure 11a for *R* = 8 and *M* = 96. Better harmonics characterization can be achieved by giving each nucleotide position within the window a relative weight in contrast to the rectangular where equal weighting is given to all nucleotides. It turns out that the Blackman window has the best main-to-first side lobe attenuation behavior as shown in Figure 11b compared to the rectangular window case and therefore provides the best smoothing of the DNA spectrum.

**Table 3:** **FIR window Specifications: relative peak side lobe *A***_**1**_**/*A***_**0**_**in dB, approximate width of main lobe Δ*ω*, equivalent Kaiser window coefficient *β*, and transition width Δ*ω***_***β***_.

**FIR Window**	***A***_**1**_**/*A***_**0**_	**Δ*ω***	***β***	**Δ** ***ω***_***β***_
Rectangular	−13	4*Π*/(*M*/*R* + 1)	0	1.81*ΠR*/*M*
Bartlett	−25	8*ΠR*/*M*	1.33	2.37*ΠR*/*M*
Hanning	−31	8*ΠR*/*M*	3.86	5.01*ΠR*/*M*
Hamming	−41	8*ΠR*/*M*	4.86	6.27*ΠR*/*M*
Blackman	−57	12*ΠR*/*M*	7.04	9.19*ΠR*/*M*

By replacing **h** with $\tilde{h}$, the matrix **H** can be in turn expressed as

$$\begin{array}{c} \tilde{H}=\left[\begin{array}{ccc} h_{0}h_{1}‥h_{M/R-1} & \cdots & 0\;0‥0\\ \vdots & \ddots & \vdots \\ 0\;0.0 & \cdots & \underset{M/R}{\underset{︸}{h_{0}h_{1}\;..\;h_{M/R-1}}} \end{array}\;\right], \end{array}$$

and the complex row vector $C^{T}\tilde{H}$ is now given by

$$\begin{array}{c} C^{T}\tilde{H}=\left[\underset{M/R}{\underset{︸}{h_{0}\;..\;h_{\frac{M}{R}-1}}}\;\ldots \;\underset{M/R}{\underset{︸}{h_{0}e^{-j2\Pi \frac{R-1}{R}}\;..\;h_{\frac{M}{R}-1}e^{-j2\Pi \frac{R-1}{R}}}}\right]. \end{array}$$

It can be easily seen that the sum of elements in $C^{T}\tilde{H}$ is still equal to zero as was the case for **C**^*T*^**H**. Consequently, it follows that the sum of any row or column in $\tilde{W}={\tilde{H}}^{H}D\tilde{H}$ is still equal to zero. This is a fundamental result which, in turn, implies that all the derivations of Section 3 are still the same even when $\tilde{h}$ replaces **h**. In particular, the -based DNA spectrum ${\tilde{S}}_{v}(n)$ and the -based one ${\tilde{S}}_{d}(n)$ can be stated as

(36) $$\begin{array}{ll} \;{\tilde{S}}_{v}(n) & ={\hat{x}}_{v}^{H}(n)(I_{4}⊗\tilde{W}){\hat{x}}_{v}(n),\;{\tilde{S}}_{d}(n)\\ & ={\hat{x}}_{v}^{H}(n)(B⊗\tilde{W}){\hat{x}}_{v}(n). \end{array}$$

Moreover, all the mathematical relations derived in Section 3 between the -based spectrum and the Voss-based one are all still valid even when **h** is replaced by $\tilde{h}$.

#### Experimental verification

**Figure 12:**
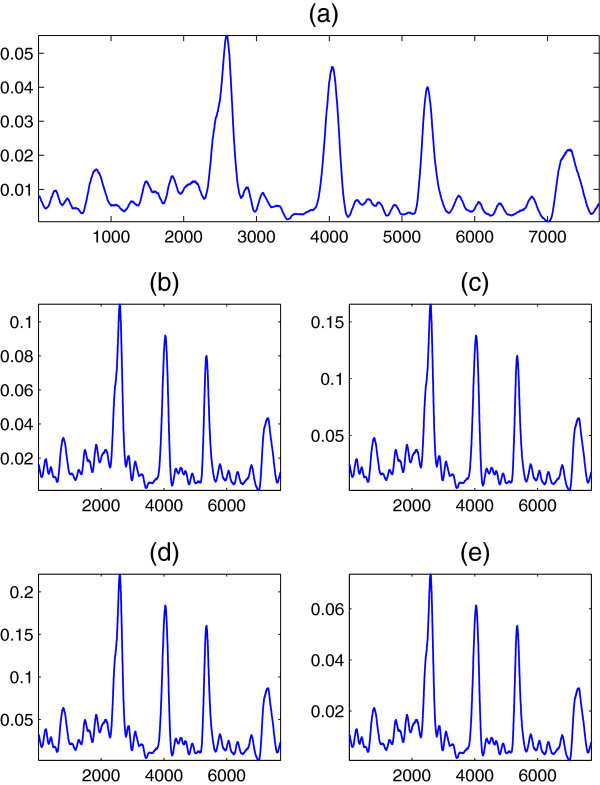
**DNA spectrum**${\tilde{S}}_{d}(n)$**with**$\tilde{h}$**set to a Blackman window at *R *** **= 3 of the DNA sequence F56F11.4 when (a) Voss, (b) tetrahedral, (c) quaternion, (d) *Z*-curve, and (e) simplex mappings are used.**

**Figure 13:**
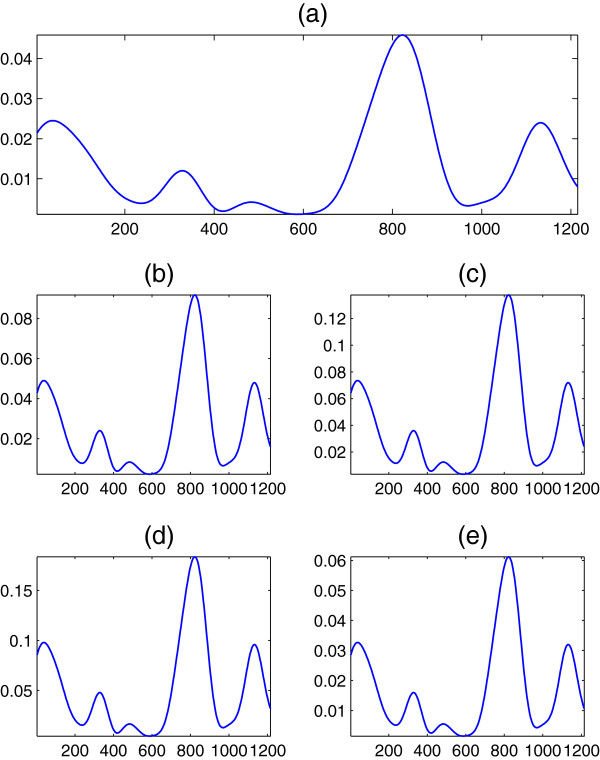
**DNA spectrum**${\tilde{S}}_{d}(n)$**with**$\tilde{h}$**set to a Blackman window at *R *** **= 3 of the DNA sequence ECZGL2 when (a) Voss, (b) tetrahedral, (c) quaternion, (d) *Z*-curve, and (e) simplex mappings are used.**

**Figure 14:**
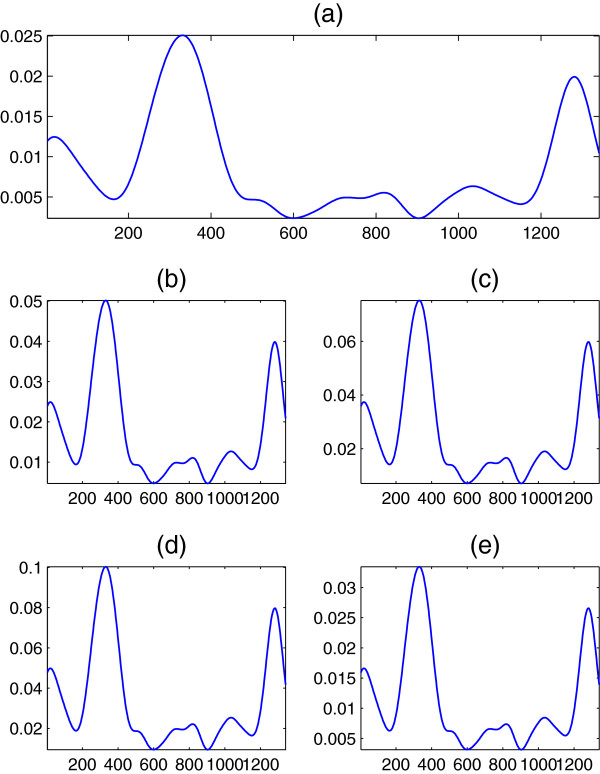
**DNA spectrum**${\tilde{S}}_{d}(n)$**with**$\tilde{h}$**set to a Blackman window at *R *** **= 3 of the DNA sequence ALOEGLOBIM when (a) Voss, (b) tetrahedral, (c) quaternion, (d) *Z *-curve, and (e) simplex mappings are used.**

To experimentally verify this result, we consider finding the DNA spectrum ${\tilde{S}}_{d}(n)$ of the three DNA sequences used in the previous section when $\tilde{h}$ is set to a Blackman window. The relations between the spectra when using the Voss, tetrahedral, quaternion, Z-curve, and simplex mappings are still the same as shown in Figures 12, 13, and 14.

#### 5.1.2 Updating the complex filter **C**

Similar to *H*(*z*), the FIR filter *C*(*z*) can be replaced by a more sophisticated filter $\tilde{C}(z)$ expressed as

$$\begin{array}{c} \tilde{C}(z)=C_{0}+C_{1}z^{-1}+\cdots +C_{R-1}z^{-(R-1)}, \end{array}$$

which is the -transform of the general array $\tilde{C}$ given by

$$\begin{array}{c} \tilde{C}=\left[C_{0}\;C_{1}\;\ldots \;C_{R-1}\right]. \end{array}$$

Note that, in this case, the elements in array $\tilde{C}$ do not necessarily add to zero anymore. Consequently, the sum of elements in any row or any column in $\tilde{D}={\tilde{C}}^{⋆}{\tilde{C}}^{T}$ or $\tilde{W}=H^{H}\tilde{D}H$ is not necessarily zero. We also note that unlike the case of $\tilde{h}$, using $\tilde{C}$ instead of **C** keeps the spectrum formulas in (58) correct but does not preserve the mathematical relations between the different -mapped spectra and the Voss-based spectrum.

#### 5.1.3 Joint optimization of $\tilde{h}$ and $\tilde{C}$

It should be clear at this point that better DNA harmonics detection performance can be potentially achieved through a joint “optimization” of $\tilde{h}$ and $\tilde{C}$. For example, a learning paradigm can be used with a least-mean-square (LMS) criterion to find the optimal set, $\tilde{h}$ and $\tilde{C}$. Alternatively, a biologically induced criterion can yield a substantial boost in performance but it is not clear which criterion to use. This interesting but challenging research topic is however outside the scope of this article and will not be further pursued here.

##### Example

**Figure 15:**
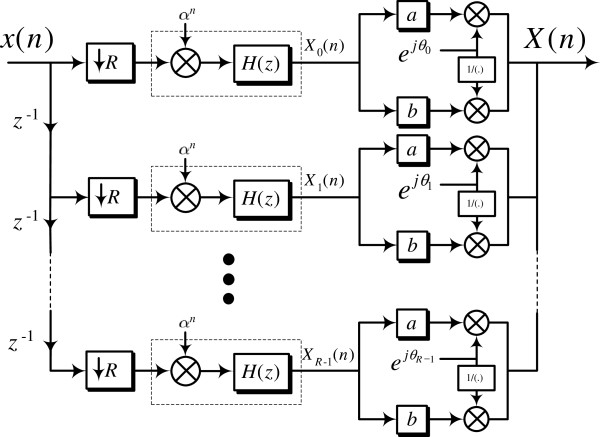
A general multirate DSP structure to compute the short-time DFT, DCT, DST, and DHT.

Standard discrete time transforms have been proposed to replace the ST-DFT in the periodicity detection problem. In particular, the short time discrete cosine transform (ST-DCT), sine transform (ST-DST), and Hartley transform (ST-DHT) were introduced and analyzed for this purpose [33]. In this example, we show that these three transforms fit naturally within our proposed analysis when the two arrays $\tilde{h}$ and $\tilde{C}$ are adjusted correctly for each case. Although these standard transforms are not optimized for certain data sets, they can serve as preliminary tests for better periodicity detection. In [33], the short time DFT, DCT, DST, and DHT at *k* = *M*/*R* where shown to be given by

(37) $$\begin{array}{c} X^{\left(t\right)}(n)=\overset{R-1}{\underset{r=0}{\sum }}C_{r}^{\left(t\right)}\overset{\frac{M}{R}-1}{\underset{m=r,r+R,\ldots }{\sum }}x(n+\mathrm{mR}+r)h^{\left(t\right)}(m), \end{array}$$

where *t* ∈ {*f*,*c*,*s*,*h*} indicates Fourier, cosine, sine, and Hartley transforms, respectively, $C_{r}^{\left(t\right)}=a^{\left(t\right)}e^{j\theta_{r}^{\left(t\right)}}+b^{\left(t\right)}e^{-j\theta_{r}^{\left(t\right)}}$ are possibly complex coefficients, and *h*^(*t*)^(*m*) = (*α*^*t*^)^*m*^. Values of the parameters *α*, *a*, *b*, and *θ*_*r*_ for every transform are adjusted according to Table 4. For illustration, setting *α* = 1, *a* = 1, *b* = 0, and *θ*_*r*_ = −2*Πr*/*R* in (59) results in the ST-DFT case. An efficient implementation to calculate Equation (59) is shown in Figure 15 which generalizes Figure 3.

**Table 4:** **Parameter settings in Figure **15** to compute the short time Fourier, cosine, sine, and Hartley transforms**

**Transform**	***α***	**a**	**b**	***θ***_***r***_
ST-DFT	1	1	0	−2*Πr*/*R*
ST-DCT	−1	1/2	−1/2	(2*r* + 1)*Π*/2*R*
ST-DST	−1	1/2*j*	−1/2*j*	(2*r* + 1)*Π*/2*R*
ST-DHT	1	$\frac{1}{2}(1-j)$	$-\frac{1}{2}(1-j)$	2*Πr*/*R*

This model provides a general framework that encapsulates the computation of the short-time Fourier, cosine, sine, and Hartley transforms at frequency point *k* = *M*/*R*. Therefore, the same matrix-based analysis of Sections 2 and 3 can be used. Matrix **W** will be updated into

$$\begin{array}{lcl} \tilde{W}={\tilde{H}}^{H}\tilde{D}\tilde{H}={\left(I_{R}⊗{\tilde{h}}^{T}\right)}^{H}{\tilde{C}}^{⋆}{\tilde{C}}^{T}\left(I_{R}⊗{\tilde{h}}^{T}\right), & & \end{array}$$

and therefore the -based DNA spectrum ${\tilde{S}}_{d}(n)$ when one of the ST- DFT, DCT, DST, or DHT is employed can be stated as

(38) $$\begin{array}{c} {\tilde{S}}_{d}(n)={\hat{x}}_{v}^{H}(n)(B⊗\tilde{W}){\hat{x}}_{v}(n), \end{array}$$

where the values of $\tilde{h}$ and $\tilde{C}$ are adjusted according to Table 5.

**Table 5:** **Modified arrays**$\overset{\sim }{h}$**and**$\overset{\sim }{C}$**to compute the short time Fourier-, cosine-, sine-, and Hartley-based DNA spectrum of (60)**


ST-DFT	$\tilde{h}=h=\{{\left(1\right)}^{i},i=1,2,\ldots M/R\}$
	$\tilde{C}=C=\{e^{-j2\Pi r/R},r=1,2,\ldots R\}$
ST-DCT	$\tilde{h}=\{{\left(-1\right)}^{i},i=1,2,\ldots M/R\}$
	$\tilde{C}=\{\cos((2r+1)\Pi /2R),r=1,2,\ldots R\}$
ST-DST	$\tilde{h}=\{{\left(-1\right)}^{i},i=1,2,\ldots M/R\}$
	$\tilde{C}=\{\sin((2r+1)\Pi /2R),r=1,2,\ldots R\}$
ST-DHT	$\tilde{h}=h=\{{\left(1\right)}^{i},i=1,2,\ldots M/R\}$
	$\tilde{C}=\{\cos(2\Pi r/R)+\sin(2\Pi r/R),r=1,2,\ldots R\}$

Note that similar to the Fourier case, the sum of elements in $\tilde{C}$ for the cosine and Hartley transforms cases is equal to zero. Therefore, under these two cases, the relations between different -based DNA spectra and the -based DNA spectrum are still the same as given in Section 3.

**Figure 16:**
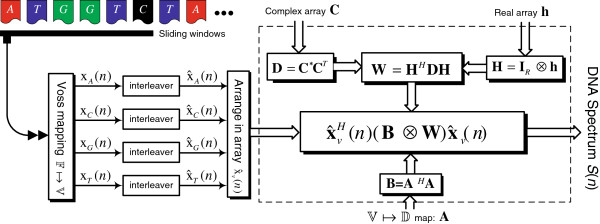
**A DSP structure to compute the modified****-based DNA spectrum **${\tilde{S}}_{d}(n)$**.** The Voss-based array of interleaved windows ${\hat{x}}_{v}(n)$, the V↦D mapping matrix **A**, the real array $\tilde{h}$, and the generally complex array $\tilde{C}$ are the system design parameters.

At this point, it can be concluded that the -based DNA spectrum ${\tilde{S}}_{d}(n)$ is completely defined in terms of the Voss-based array of interleaved windows ${\hat{x}}_{v}(n)$, the V↦D mapping matrix **A**, the real array $\tilde{h}$, and the generally complex array $\tilde{C}$. This conclusion is summarized in Figure 16.

### 5.2 A real approach for the spectrum computation

A real computationally-efficient alternative for the evaluation of *S*_*d*_(*n*) can be found by observing the special properties of the circulant/toeplitz matrix **D** or equivalently the block matrix **W**. We use the fact that for a generally-complex matrix **Q**: *y*^*H*^**Q***y* = 0, ∀y∈R, if **Q** is an antisymmetric matrix. We start by splitting **D** into its symmetric and antisymmetric parts

$$\begin{array}{c} D=\underset{\text{symmetric}}{\underset{︸}{\frac{1}{2}\left(D+D^{T}\right)}}+\underset{\text{antisymmetric}}{\underset{︸}{\frac{1}{2}\left(D-D^{T}\right)}}=D_{s}+D_{\mathrm{as}}, \end{array}$$

where **D**_*s*_ is a circulant and Toeplitz real *R* × *R* matrix given by

$$\begin{array}{c} D_{s}=\left[\begin{array}{cccc} 1 & 2\cos\frac{2\Pi }{R} & \cdots & 2\cos\frac{2\Pi (R-1)}{R}\\ 2\cos\frac{2\Pi }{R} & 1 & \ddots & \vdots \\ \vdots & \ddots & \ddots & 2\cos\frac{2\Pi }{R}\\ 2\cos\frac{2\Pi (R-1)}{R} & \cdots & 2\cos\frac{2\Pi }{R} & 1 \end{array}\right], \end{array}$$

and **D**_*as*_ is a circulant and Toeplitz complex *R* × *R* matrix given by

$$\begin{array}{c} D_{\mathrm{as}}=2j\left[\begin{array}{cccc} 0 & -\sin\frac{2\Pi }{R} & \cdots & -\sin\frac{2\Pi (R-1)}{R}\\ \sin\frac{2\Pi }{R} & 0 & \ddots & \vdots \\ \vdots & \ddots & \ddots & -\sin\frac{2\Pi }{R}\\ \sin\frac{2\Pi (R-1)}{R} & \cdots & \sin\frac{2\Pi }{R} & 0 \end{array}\right]. \end{array}$$

Substituting for **D** in (15), we get a simple form of the spectrum *S*(*n*)

(39) $$\begin{array}{ll} \;S(n) & ={\hat{x}}^{H}(n)\;H^{H}DH\;\hat{x}(n)\\ & ={\hat{x}}^{H}(n)\;H^{H}\left(D_{s}+D_{\mathrm{as}}\right)H\;\hat{x}(n)\\ & ={\hat{x}}^{H}(n)\;W_{s}\;\hat{x}(n), \end{array}$$

where $y^{H}D_{\mathrm{as}}y=0,\forall \;l\in F$, $y=H\;\hat{x}(n)$. The block matrix

$$\begin{array}{c} W_{s}≐H^{H}D_{s}H=\frac{1}{2}H^{H}\left(D+D^{T}\right)H \end{array}$$

is an *R* × *R* matrix of $\frac{M}{R}\times \frac{M}{R}$ blocks. Using (63) to update the DNA spectrum (21), *S*_*v*_(*n*) simplifies into

$$\begin{array}{c} S_{v}(n)=\underset{l\in F}{\sum }{\hat{x}}_{l}^{H}(n)\;W_{s}\;{\hat{x}}_{l}(n). \end{array}$$

Following the same analysis of Section 3, (64) can be easily manipulated into a more elegant completely real form given by

(40) $$\begin{array}{c} S_{v}(n)={\hat{x}}_{v}^{H}(n)\left(I_{4}⊗W_{s}\right){\hat{x}}_{v}(n), \end{array}$$

or more generally, (35) can be updated into

(41) $$\begin{array}{c} S_{d}(n)={\hat{x}}_{v}^{H}(n)\left(B⊗W_{s}\right){\hat{x}}_{v}(n), \end{array}$$

which provides a completely real approach for the computation of the -mapped spectrum *S*_*d*_(*n*). Note that all results and different spectra relations in Section 3 still hold when **W**_*s*_ replaces **W** as in (65).

#### Computational complexity comparison

To quantify the computational credit of this real approach, we compare the complexity of (63) to that of (16) of a single discrete time sequence. Since $\hat{x}(n)$ can be complex as well according to the mapping used, we find the number of real multiplications and additions needed to evaluate (63) when each of $\hat{x}(n)$ and **W** is either real or complex, as given in Table 6. Recall that the multiplication of the complex numbers *x* and *y*, where *x* = *a* + *jb* and *y* = *c* + *jd* requires the computation of *ac* − *bd* and *ad* + *bc*, which requires four real multiplications and two real additions.

**Table 6:** Real multiplications and additions needed for the evaluation of (63) and (16)

$\hat{x}(n),W$	**Real multiplications**	**Real additions**
real,real	*M*(*M* + 1)	*M*^2^ − 1
real,complex	2*M*(*M* + 1)	2(*M*^2^ − 1)
complex,real	2*M*(*M* + 1)	2(*M*^2^ − 1)
complex,complex	4*M*(*M* + 1)	2(2*M*^2^ + *M* − 1)

#### Example

For illustration, we evaluate the spectrum *S*_*v*_(*n*) using **W**_*s*_ when *R* = 3, and compare the result to the formula derived in [37]. In specific, we use (64) to find the spectrum *S*(*n*) as follows

$$\begin{array}{ll} \;S_{v}(n) & =\underset{l\in F}{\sum }{\hat{x}}_{l}^{H}(n)H^{H}D_{s}H{\hat{x}}_{l}(n)=\underset{l\in F}{\sum }\Gamma_{l}^{H}(n)D_{s}\;\Gamma_{l}(n)\\ & =\underset{l\in F}{\sum }[X_{l0}X_{l1}X_{l2}]\;\left[\begin{array}{ccc} 1 & -1 & -1\\ -1 & 1 & -1\\ -1 & -1 & 1 \end{array}\right]\;\left[\begin{array}{c} X_{l0}\\ X_{l1}\\ X_{l2} \end{array}\right]. \end{array}$$

Expanding and completing the square, it follows that

(42) $$\begin{array}{ll} \;S_{v}(n) & =\underset{l\in F}{\sum }[X_{l0}^{2}(n)+X_{l1}(n)(X_{l1}(n)-X_{l0}(n))\\ & +X_{l2}(n)(X_{l2}(n)-X_{l0}(n)-X_{l1}(n))]\\ & =\frac{1}{2}\underset{l\in F}{\sum }\overset{2}{\underset{r=0}{\sum }}{\left(X_{\mathrm{lr}}(n)-X_{\mathrm{lq}}(n)\right)}^{2}, \end{array}$$

where *q* = (*r* + 1) mod 3. The matrix-based DNA spectrum formula in (67) is consistent with the result derived using a different approach in [37].

## 6 Concluding remarks

In this article, we have introduced a matrix based framework for locating hidden DNA periodicities using spectral analysis techniques that are invariant to the choice of the symbolic to numerical map. The primary advantage of the presented approach over some of the previous study is the decomposition of the spectrum expression into key matrices whose values can be set *independently from each other*. Each matrix represents one of the essential components involved in the computation of the spectrum such as the symbolic to numerical map, the data transform, and the filtering scheme. The above framework is derived under the assumption that the symbolic to numerical map can be obtained from the Voss representation using an affine transformation. This assumption is however quite loose given that most (if not all) of the proposed maps in the literature satisfy this requisite. Using the new framework, we have then shown that the DNA spectrum expression is invariant under these maps. We have also derived a necessary and sufficient condition for the invariance of the DNA spectrum in terms of the affine transformation matrix **A** (the **b** vector in the affine transformation does not affect the DNA spectrum).

This condition can serve as the basis for generating novel symbolic to numerical map that preserve the DNA spectrum expression. Finally, in the latter sections of the article, we have shown the potential of using different filtering schemes, e.g., windows other than the rectangular one as well as alternate fast data transforms, e.g., the DCT, DST, and the Hartley transform. A number of simulation results that verify the findings of this article and a brief quantitative analysis of the computational complexity of the new approach were given in the same sections. Future research study would consider the optimization of the different building blocks, namely the symbolic to numerical map, the data transform, and the filtering scheme. This, in turn, requires a deep understanding of the biological significance of different DNA periodicities in order to set up a meaningful objective function and appropriate constraints. Ultimately, the framework proposed here can be incorporated in a more sophisticated system to study the complex structure of genomic sequences and understand the functionality of its various components. Finally, this efficient framework can be extended to the analysis of other types of symbolic sequences of various limited alphabets, either biological sequences (such as protein sequences) or even non-biological ones.

## Competing interests

The authors declare that they have no competing interests.
